# Learning rate and temperament in a high predation risk environment

**DOI:** 10.1007/s00442-014-3099-z

**Published:** 2014-10-02

**Authors:** C. DePasquale, T. Wagner, G. A. Archard, B. Ferguson, V. A. Braithwaite

**Affiliations:** 1 Department of Biology, Pennsylvania State University-Altoona, 207 Hawthorn Building, 3000 Ivyside Park, Penn State-Altoona, Altoona, PA 16601 USA; 2 Center for Brain, Behavior, and Cognition, Pennsylvania State University, University Park, PA USA; 3 US Geological Survey, Pennsylvania Cooperative Fish and Wildlife Research Unit, Pennsylvania State University, University Park, PA USA; 4 School of Biological Sciences, Cardiff University, Cardiff, UK; 5 Department of Ecosystem Science and Management, Pennsylvania State University, University Park, PA USA

**Keywords:** Predation pressure, Temperament, Boldness, Associative learning, Conditioning

## Abstract

Living in challenging environments can influence the behavior of animals in a number of ways. For instance, populations of prey fish that experience frequent, nonlethal interactions with predators have a high proportion of individuals that express greater reaction to risk and increased activity and exploration—collectively known as temperament traits. Temperament traits are often correlated, such that individuals that are risk-prone also tend to be active and explore more. Spatial learning, which requires the integration of many sensory cues, has also been shown to vary in fish exposed to different levels of predation threat. Fish from areas of low predation risk learn to solve spatial tasks faster than fish from high predation areas. However, it is not yet known whether simpler forms of learning, such as learning associations between two events, are similarly influenced. Simple forms of associative learning are likely to be affected by temperament because a willingness to approach and explore novel situations could provide animals with a learning advantage. However, it is possible that routine-forming and inflexible traits associated with risk-prone and increased exploratory behavior may act in the opposite way and make risk-prone individuals poorer at learning associations. To investigate this, we measured temperament in Panamanian bishop fish (*Brachyrhaphis episcopi*) sampled from a site known to contain many predators. The *B. episcopi* were then tested with an associative learning task. Within this population, fish that explored more were faster at learning a cue that predicted access to food, indicating a link between temperament and basic learning abilities.

## Introduction

The ability of animals to learn varies among individuals, and understanding what generates such variation has been central to the developing field of cognitive ecology (Dukas 2009). Numerous studies have demonstrated the important role that experience during development can play in terms of shaping adult perception, as well as learning and memory processes (Wiltschko et al. 1989; Huntingford 2004; Woolley 2012). For free-living animals, this means that local conditions can influence how both behavior and cognitive skills develop (Healy and Braithwaite 2000). Within evolutionary ecology, one variable known to have a major effect on populations from different geographic locations is exposure to predation threat (Endler 1995). Prey species living in areas of high or low predation risk have contrasting experiences that result in a suite of traits that clearly differentiate the populations. For example, life-history traits of populations that experience contrasting levels predation threat differ, such that animals with a higher risk of predation tend to become reproductively mature at a smaller size (Reznick and Endler 1982; Lafferty 1993; Jennions and Telford 2002). Predator avoidance responses, such as living in groups, and predator inspection behaviors also become more refined in individuals that need a higher awareness of threats within the local environment (Magurran 1990; Magurran and Seghers 1990).

Increasingly, behavioral studies are addressing the role of temperament in animal behavior (Réale et al. 2007; Sinn et al. 2008; Smith et al. 2009). Temperament describes different behavioral traits such as aggression, boldness (an individual’s reaction to risky, non-novel situations), exploration (willingness to respond to a novel situation), and activity. Exposure to different developmental experiences and environmental conditions contribute to shaping the development of certain temperament traits (for a review, see Réale et al. 2007). For example, predation pressure has been shown to influence temperament in three-spined sticklebacks (*Gasterosteus aculeatus*); consistent correlations between aggression, activity, and exploration were found in fish living in high-predation locations, but not in fish where predators were absent (Dingemanse et al. 2007). Fish from high-predation locations that were relatively aggressive were also more active and exploratory compared to less aggressive fish.

The degree to which an animal expresses tendencies to be risk-prone or exploratory is likely to influence aspects related to learning and memory. In risky situations, such as exposure to predators, we might predict that more risk-prone animals would have a learning advantage because they would move over larger areas and likely discover changes to their environment more rapidly. Interestingly, where this has been explored, the opposite appears to occur (Coppens et al. 2010). For instance, a study with pigs showed that individuals which expressed more bold-like behaviors had poorer performance in a spatial learning task (Bolhuis et al. 2004). Similarly, in comparison to more timid individuals from low-predation sites, bold fish from high-predation areas took longer to learn the location of food and shelter in a multi-patch environment where only one patch contained accessible food and shelter (Brown and Braithwaite 2005). It is well known that bolder individuals tend to form relatively inflexible routines (Sih and Del Giudice 2012). Therefore, although learning can allow animals to adjust their behavior through experience (Antunes and Oliveira 2009), animals that have more risk-prone, bolder temperament traits appear to be constrained in terms of their learning capacity and their degree of behavioral flexibility (Sih et al. 2004; Bergmüller 2010).

Many of the studies investigating the relationship between learning and temperament have tended to focus on more complex tasks which depend on the integration of many sensory cues, such as spatial or social learning (Brydges et al. 2008; Kurvers et al. 2010). Much less attention has focused on simple forms of learning where animals learn to form associations such that one event is linked with or can predict something about another event (Pearce 1997). In this way, associative learning can help animals to anticipate when certain events or processes will occur. For example, male blue gourami (*Trichogaster trichopterus*) can learn to associate a particular cue with the arrival of a female in their territory. After learning that the cue predicts the arrival of a female, the males quickly switch from territory defense behaviors to courtship, which results in higher mating success in comparison to poor learners (Hollis et al. 1997). Fish can also learn associations to avoid negative situations; rainbow trout (*Oncorhynchus mykiss*) can learn to predict when conspecifics will be aggressive and can use this to preemptively escape faster (Carpenter and Summers 2009). A number of studies have also shown that fish can be trained to predict when food will be available and they will approach a feeding area shortly after a light is switched on (Atlantic cod, *Gadus morhua*: Nilsson et al. 2008a, b; rainbow trout: Nordgreen et al. 2010; Atlantic salmon: Bratland et al. 2010).

We might expect that animals which actively move around their environment and approach or explore more will encounter changes in the environment sooner than individuals that move less. Sneddon (2003) found that active rainbow trout were faster at learning to associate a light switching on with food being available compared to less active conspecifics. Therefore, individuals that have higher exploratory and more risk-prone tendencies may have better associative learning abilities.

To investigate how simple learning is influenced by temperament traits in a natural context, we chose to work with individual Panamanian bishop fish (*Brachyrhaphis episcopi* Steindachner) from a population where there is a high risk of predation. Previous research has shown that individuals from this population tend to express greater levels of exploration (Archard and Braithwaite 2011). Here, we investigated how quickly fish learn to associate a light cue with access to food. We predicted that fish which showed higher levels of exploration would learn at a slower rate than less exploratory individuals, as this is typically seen in animals that form inflexible routines, and pay less attention to changes in their environment.

## Methods

Thirty female *B. episcopi*, a small, live-bearing, poeciliid fish endemic to Panama, were captured using seine and hand nets in the lower reaches of the Rio Macho (09°10.932′N; 079°45.674′W). The site was selected as it contains multiple species of predatory fish that prey on *B. episcopi* (Brown et al. 2005). The fish were transported to the Smithsonian Tropical Research Institute field station in Gamboa, where they were placed in 50-l holding tanks and kept on a 12:12 light:dark cycle. The tanks contained a filter and aeration, and water was maintained at 25–26 °C at a depth of approximately 30 cm. They were allowed 3 days to acclimate and to ensure that they readily fed on commercial flake food.

Exploration and activity were assessed using an open field test—a method previously validated to quantify reliable temperament traits in this species (Archard and Braithwaite 2011). Previous work on wild *B. episcopi* has also shown that open field behaviors are repeatable in this species (G.A. Archard, V.A. Braithwaite, and N. Colegrave, unpublished data). The trials work by placing an individual in a novel open arena which has no escape opportunity, and the response of the animal to this environment is monitored (Walsh and Cummins 1976). An opaque, plastic test tank (length 40 cm × width 30 cm × height 24 cm) filled to a depth of 10 cm with freshwater was set up with a video camera suspended centrally above it to allow trials to be filmed. The tank was positioned in such a way that the lighting was even across the whole tank. The base of the tank was marked with 7 by 7 cm squares, with bolder lines highlighting the outer (the outermost 7-cm squares around the perimeter) and inner sections. The sides of the arena were covered with black plastic to minimize disturbance from any movement outside the tank.

At the start of a trial, an individual fish was carefully moved from the home tank using a dip net and placed in a transparent start cylinder (diameter, 5 cm) placed in the middle of the test tank. After 2 min of acclimatization, video recording was started and the cylinder was slowly raised remotely so that the fish was free to explore the test tank. Trials lasted 5 min before the fish was removed using a dip net. The videos of the open field test were analyzed using Etholog v2.2.5 (Ottoni 2000). Ethology was used to measure when the fish was moving or frozen, and when it crossed a line on the grid marked on the bottom of the arena. These data were then used to calculate rate of movement (number of lines crossed/min), propensity to freeze (proportion of the 5-min trial spent frozen), and time taken to reach the edge of the arena (after the start cylinder was removed), which have previously been shown to be reliable measures of exploration and activity (Archard and Braithwaite 2011; Archard et al. 2012).

To test the hypothesis that temperament traits influence acquisition of a simple associative task, five experimental tanks (length 36 cm × width 18 cm × height 28 cm) were each subdivided into three compartments (length 12 cm × width 18 cm × height 28 cm) using sheets of netting fabric secured in place with silicon sealant. An individual fish was placed in each compartment and remained there for the remainder of the study. This allowed individual learning performance to be tracked, while permitting the fish some visual contact with conspecifics. Although it is possible that olfactory cues from fish in adjacent compartments had an effect on learning, we attempted to keep this at a minimum by replacing a third of the water in the tank with clean aquarium water every day. A plastic box filter was placed in the middle compartment of each tank, and similar-sized, upturned half plant pots were placed in the two outer compartments so that each compartment was similarly furnished. A floating food ring (diameter, 6 cm) was attached to the front wall of each compartment at the water’s surface. The top of each tank was covered with a layer of netting to prevent the fish from escaping. To give the human observer physical access to the tank to deliver flake food, holes were cut in the netting directly over the food ring. Another piece of netting was placed over this to ensure the food holes were covered when trials were not taking place.

The fish were given 2 days to acclimate to the compartments, and during this time their food was delivered in the food ring. During the conditioning trial period, sheets of opaque laminated card were placed between each compartment (or tank) 30 min before a training trial was given so that the fish were visually isolated during the trials. A delay-conditioning task was used to monitor rate of learning. This was achieved using a flashlight that was shone directly above the food ring for 12 s, before flake food was delivered into the food ring. The light remained on for an additional 12 s before it was switched off (an overlap of 12 s). Successful conditioning could be seen when the fish responded to the onset of the light cue by moving toward the food ring; at this stage the fish was considered to have demonstrated a learned association.

Training trials were given twice a day for 7 days (one trial in the morning and one in the afternoon). Following Sneddon (2003), a fish was considered to have learned after successfully performing three consecutive trials where the fish showed a conditioned response (CR), approached within one body length of the food ring within 12 s or less of the light being switched on, and then fed within 24 s (i.e., before the light was switched off). Thus, for each fish, “success” was quantified at the end of three consecutive trials in which the fish demonstrated the learned light–food association. For instance, an individual that consecutively displayed a learned response during trials 12, 13, and 14 would be given a score of 14. Three fish froze during the training trials and failed to reach the CR criterion after 14 trials, so they were excluded from the analyses.

All data met assumptions of equality of variance and normality. The prediction was that exploration and activity in the open field trial would be related to the rate at which a fish would learn the conditioning task. This was tested using simple linear regression with number of trials to learn in three consecutive trials as the response variable and different open field test measures as the predictor variables. Linear regression with multiple predictor variables was also carried out to determine which open field measures best explained the variation in learning rates. Separate models were used when predictor variables showed high correlation (*r* > ±0.60). Although we acknowledge that multiple testing can influence type I errors, we had two main goals before the experiment: to describe how each independent predictor variable influenced learning, and to describe how much variation in learning was explained by all the predictors together. All analyses were performed using R (R Development Core Team 2010) and the level of significance was set at *α* = 0.05.

## Results

**Fig. 1:**
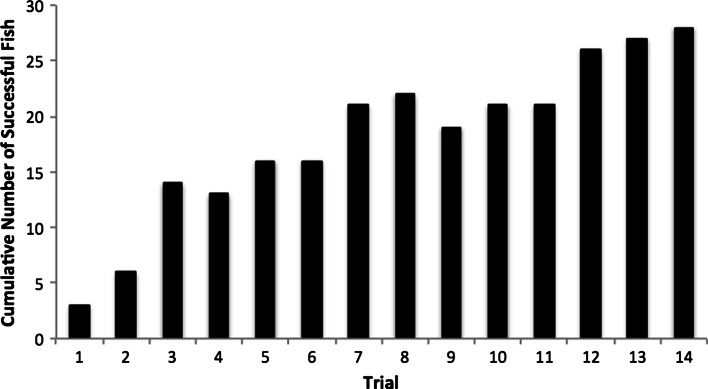
Variation in performance of the associative learning task, shown as the cumulative number of fish that successfully performed the task across trials

**Fig. 2:**
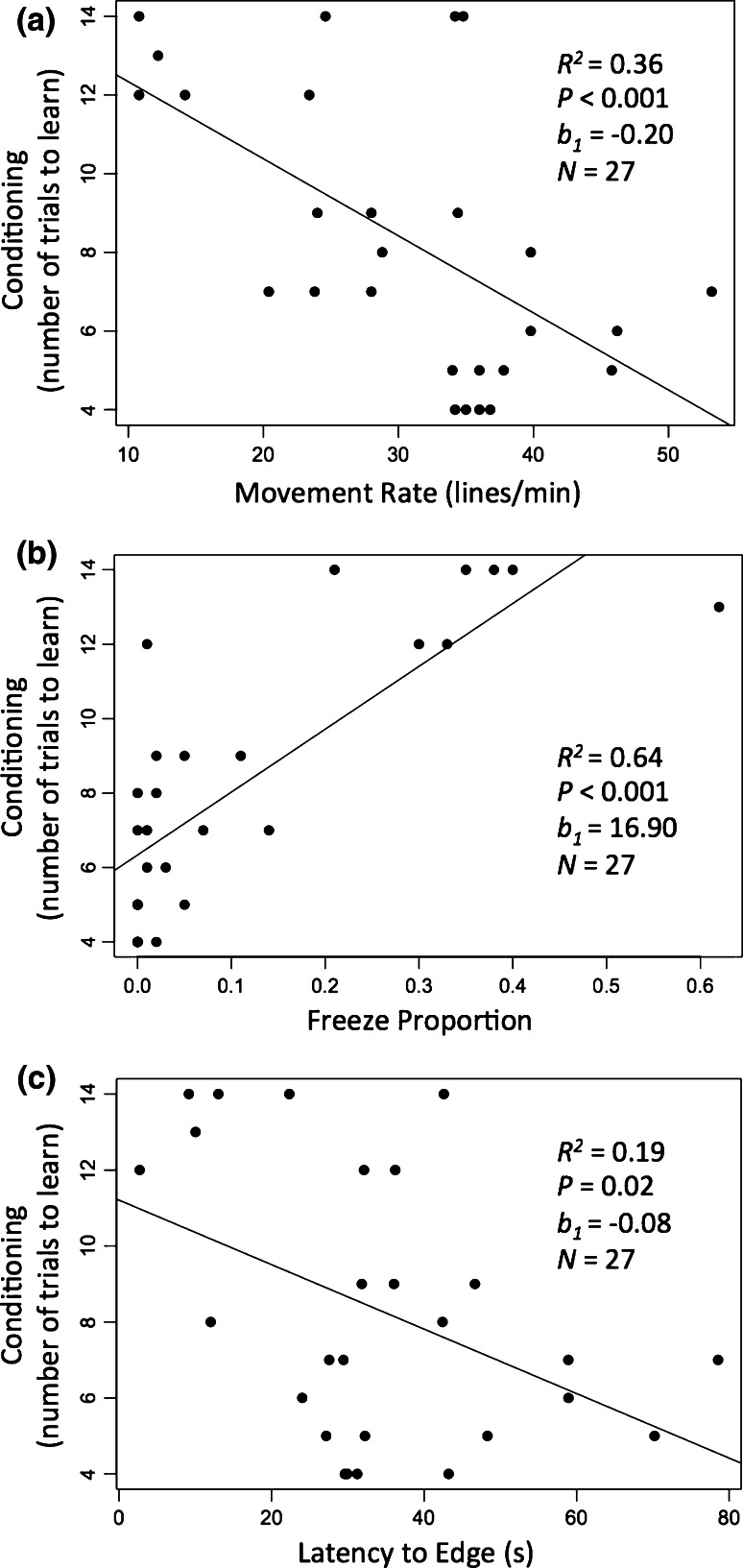
Number of trials needed to condition plotted against three different measures from the open field test: **a** movement rate, **b** proportion of time spent frozen, and **c** latency to reach the edge of the arena

The fish showed considerable individual variation in the rates at which they learned the delayed conditioning task; some took only one or two trials to learn the association between the light cue and the food reward, whereas others took more than ten trials (Fig. 1). Fish temperament was correlated with conditioning ability—individuals that were more exploratory and active in the open field test learned the conditioned response more quickly. Specifically, fast learners moved more quickly (*F*_1,25_ = 14, *P* < 0.001, *R* ^2^ = 0.36, *b*_1_ = −0.20; Fig. 2a), spent less time frozen (*F*_1,25_ = 43.79, *P* < 0.001, *R* ^2^ = 0.64, *b*_1_ = 16.90; Fig. 2b), and took longer to reach the outer zone of the arena (*F*_1,25_ = 5.82, *P* = 0.02; *R* ^2^ = 0.19, *b*_1_ = −0.08; Fig. 2c) during open field trials.

Both proportion of time spent frozen and movement rate explained more variance in the learned conditioning results than latency to the outer zone (movement rate vs. latency to outer zone, *F*_2,24_ = 9.46, *P* < 0.001, *t* = −3.29, *P* = 0.003, *t* = −1.88, *P* = 0.073; proportion of time frozen vs. latency to outer zone, *F*_2,24_ = 21.57, *P* = 4.353, *t* = 5.52, *P* < 0.001, *t* = −0.63, *P* = 0.53). Movement rate and proportion of time frozen were significantly correlated (*r* = −0.63, *P* < 0.001) and so could not be put in the same model.

## Discussion

Temperament measures quantified in an open field test were related to how quickly fish learnt to form an association between two cues. Contrary to our predictions, fish with stronger tendencies for exploration and activity learned a simple light–food association more quickly than fish that were slower and explored less. These observations contrast with those noted in an earlier study of *B. episcopi*, where a more demanding spatial task revealed that fish from timid populations are better at finding food and shelter than fish from bolder populations (Brown and Braithwaite 2005).

The ecological and evolutionary implications of temperament traits are important when considering how animals respond to natural situations such as foraging, competition, and predation, because there are costs and benefits associated with how an individual responds in a certain context (Gosling 2001; Archard and Braithwaite 2010; Carere and Locurto 2011). The idea that temperament traits can drive differences in individual learning is not a new concept in the field of cognitive ecology. However, our findings suggest that temperament traits can have differential effects on simple versus more complex forms of learning. Compared to learning associations between stimuli, a spatial task is more complex, as it requires the integration of multiple pieces of information (Pearce 1997). In comparison, learning that one cue can predict a specific event simply needs two events to be linked with one another, which is not very cognitively demanding (Pearce 1997).

Animals that behave in a bold manner are more willing to take risks in novel situations, and those that are more exploratory and active will generally experience a wider range of situations (Wilson et al. 1994; Gosling 2001). These traits could be beneficial when searching for food or seeking out mating opportunities, but at the same time they are likely to put the animal at risk in an environment frequented by predators. In the current study, more exploratory and active *B. episcopi* individuals were faster to learn that a light cue predicted delivery of a food reward, and these traits are likely to allow such individuals greater exposure to their local environment (Carere and Locurto 2011). Thus, explorative individuals could have an advantage when forming associations: if they are more willing to explore novel environments, and perhaps also to take risks, then they may have more opportunity to encounter relevant stimuli in the environment and learn associations between these.

Previous work with different species has found that more explorative individuals will sometimes form associations more quickly during a simple learning task. In birds, black-capped chickadees that were more willing to enter a novel environment were faster to learn an acoustic discrimination task than conspecifics that were less willing to enter a novel environment (Guillette et al. 2009). In addition, laboratory-reared mice (*Mus musculus*) that were more willing to explore a novel environment performed better in an associative learning task (Matzel et al. 2003, 2006). In contrast, Budaev and Zhuikov (1998) found that bolder guppies (*Poecilia reticulata*) took longer to learn an avoidance task than more timid individuals, but only when exploration activity was low. This effect was not seen in individuals with high exploration activity. Thus, individual variation in temperament seems to play a role in influencing differences in associative learning across taxa.

The way that associations form between stimuli or events can affect behaviors ranging from foraging and competition to mating (Braithwaite and Salvanes 2008). The data presented here suggest that temperament may be an important source of variation that affects learning ability. Specifically, more exploratory *B. episcopi* individuals were able to learn a simple association task more quickly. As *B. episcopi* populations from high-predation areas tend to be risk-prone, explore more, and have higher levels of overall activity than their conspecifics at low-predation sites, it seems likely that populations exposed to more predation will be better at learning simple associations (Brown et al. 2005; Archard and Braithwaite 2011; Archard et al. 2012). Thus, in a high-predation environment, being able to make rapid associations between potentially dangerous stimuli is likely to be advantageous.

The ability of wild animals to process information from the surrounding environment can be hindered when they have to divide their attention between simultaneous tasks, such as competing for a food resource and being vigilant in the presence of a predator (Dukas 2002). Fish living with predators need to focus on making strong predictions about threatening stimuli in their environment in order to survive. Thus, fish from high-predation sites may be limited in the amount of attention they can give to learning the spatial arrangement of their surroundings because their attention is diverted elsewhere. However, fish living in low predation areas can afford to divide their attention between multiple cognitive activities such that they have better spatial abilities but have less opportunities to form simpler associations in their environment. We therefore predict that populations exposed to more predation will have better associative learning skills than fish living in less dangerous environments.
